# Chemifriction and Superlubricity: Friends or Foes?

**DOI:** 10.1021/acs.jpclett.5c00193

**Published:** 2025-03-13

**Authors:** Penghua Ying, Xiang Gao, Amir Natan, Michael Urbakh, Oded Hod

**Affiliations:** † Department of Physical Chemistry, School of Chemistry, The Raymond and Beverly Sackler Faculty of Exact Sciences and The Sackler Center for Computational Molecular and Materials Science, Tel Aviv University, Tel Aviv 6997801, Israel; ‡ Department of Physical Electronics, Tel Aviv University, Tel Aviv 6997801, Israel

## Abstract

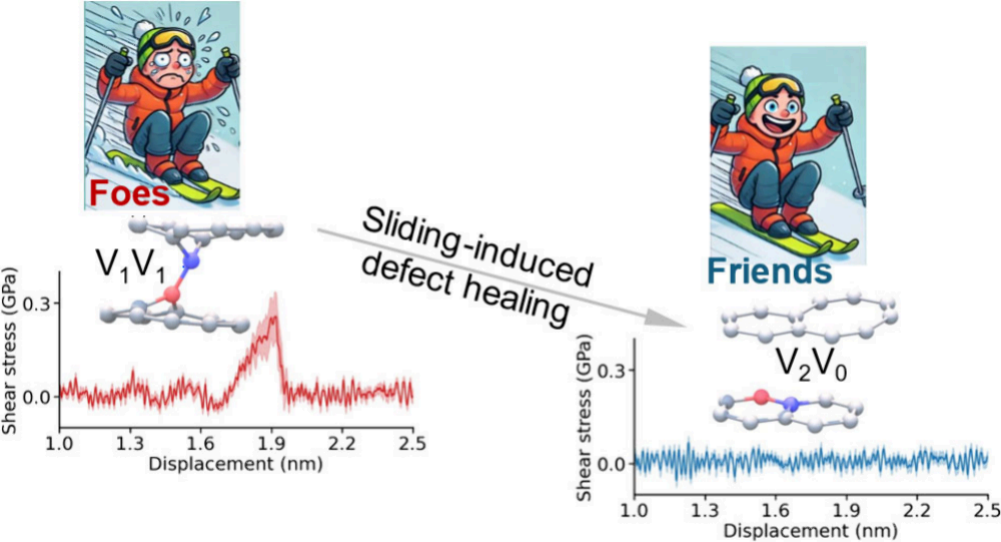

The mechanisms underlying chemifriction (the contribution of interfacial bonding to friction) in defected twisted graphene interfaces are revealed using fully atomistic molecular dynamics simulations based on machine-learning potentials. This involves stochastic events of consecutive bond formation and rupture between single vacancy defects that may enhance friction. A unique shear-induced interlayer atomic transfer healing mechanism is discovered that can be harnessed to design a run-in procedure to restore superlubric sliding. This mechanism should be manifested as negative differential friction coefficients that are expected to emerge under moderate normal loads. A physically motivated phenomenological model is developed to predict the chemifriction effects in experimentally relevant sliding velocity regimes. This allows us to identify a distinct transition between logarithmic increase and logarithmic decrease of the friction force with increasing sliding velocity. While demonstrated for homogeneous graphitic contacts, a similar mechanism is expected to occur in other homogeneous or heterogeneous defected two-dimensional material interfaces.

Sliding at the interface of two rigid, flat, and weakly interacting crystalline surfaces that are stacked in an incommensurate configuration, leads to ultralow friction.^1,2^ This is due to the effective cancellation of lateral forces opposing translational motion—a hallmark of a phenomenon referred to as structural superlubricity.^3−8^ Van der Waals (vdW) interfaces of layered contacts are ideal platforms to observe superlubricity, where interfacial incommensurability is facilitated by, e.g., the misfit angle in twisted graphitic interfaces,^9,10^ curvature effects in multiwalled carbon nanotubes,^11^ intrinsic lattice mismatch in graphene/hexagonal boron nitride heterostructure,^7,12^ and strain engineering in bilayer graphene.^13,14^ The scaling-up of superlubricity from the nanoscale to macroscopic contacts inevitably involves structural defects,^15^ such as atomic vacancies,^16,17^ steps,^18,19^ edges,^20,21^ and grain boundaries.^22,23^ The impact of such defects on the friction of superlubric sliding interfaces involves two distinct contributions: (i) physical contributions from defect-induced out-of-plane corrugation; and (ii) chemical contributions associated with the formation and rupture of interlayer covalent bonds. While the former have been extensively explored over the past decade,^24−33^ the atomistic insights into the latter remain much less understood. We recently demonstrated that this challenge can be addressed by machine-learning graph neural network potentials. Specifically, for bilayer defected graphene, we developed such a potential that achieves ab initio level accuracy in simultaneously describing sliding energy corrugation and interlayer bonding dynamics in superlubric layered contacts.^34^

In the present Letter, we use this computational framework to elucidate the phenomenon of kinetic *chemifriction*—the friction arising due to chemical bonding dynamics. We perform non-equilibrium molecular dynamics (MD) simulations to study the stochastic nature of interlayer bonding dynamics in defected twisted bilayer graphene, and the corresponding impact on the superlubric behavior of this incommensurate sliding interface. Based on the numerical results we then construct a theoretical approach that allows us to bridge between atomistic simulations and experimentally relevant time- and length-scales.

Our model system consists of a 9.43° twisted defected graphene bilayer with a single atomic vacancy defect in each layer (marked as V_1_V_1_),^35−38^ encompassing overall 590 atoms per supercell (see Figure 1a). The chosen twist angle is sufficiently large to provide a computationally accessible, laterally periodic supercell (in the absence of defects^39^) that still allows to avoid spurious interactions between periodic images of the defects. The two vacancies were positioned to ensure that upon relative sliding of 2.59 nm (half of the box length) between the top and bottom layers along the zigzag (*x*-axis) direction they align in an eclipsed configuration. Overall, the laterally periodic V_1_V_1_ model has a vacancy fraction of approximately 0.3%, comparable to experimental observations.^17^ This fundamental model system allows us to isolate, identify, and study the basic mechanisms underlying chemifriction and their interplay with physifriction, while avoiding additional complexities arising from chemical passivation or other types of defects.

**Figure 1:**
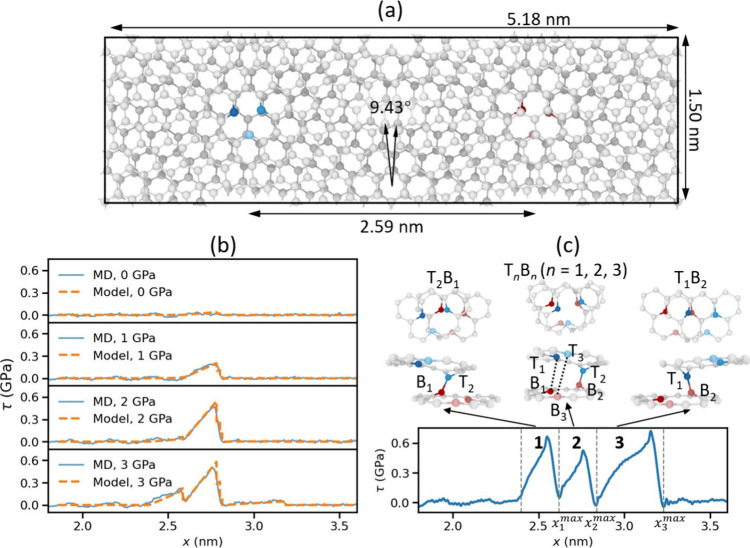
V_1_V_1_ interface sliding dynamics simulations at a temperature of 300 K and a relative sliding velocity of 10 m/s. (a) Top view of the 9.43° twisted V_1_V_1_ bilayer graphene considered in this work. The unsaturated dangling atoms around the vacancies in the top and bottom layers are highlighted in blue and red, respectively. (b) Shear stress traces of the twisted V_1_V_1_ bilayer under external normal loads of 0, 1, 2, and 3 GPa obtained from the atomistic MD simulations (solid blue lines) and the developed phenomenological model (eq 8, dashed orange lines). (c) A representative trajectory demonstrating consecutive bond formation and rupture events in three regions (marked by 1–3 and enclosed by vertical dashed lines) occurring under a normal load of 3 GPa. The friction trace (bottom) and corresponding snapshots, showing binding pathways in top (upper row) and front (middle row) views, are presented. For clarity, only atoms in the vicinity of the defects are presented. Atomistic snapshots were visualized using the OVITO package.^42^

The reactive sliding dynamics simulations were performed using the recently developed machine learning potential,^34^ as implemented in the LAMMPS package.^40^ In these simulations, the top layer is driven laterally along the zigzag direction by a rigid slider (replicating the initial top layer) via a harmonic spring, whereas the bottom layer atoms are anchored to their initial positions with springs of the same stiffness (see Supporting Information (SI) Figure S1). Due to the stochastic nature of the interlayer binding process (see, e.g., ref (41)), a minimum of 50 independent trajectories (each with a different random number seed for the initial atomic velocity distribution) was used to evaluate the bond formation probability and calculate the average kinetic friction. Unless noted otherwise, each trajectory involved a total sliding displacement of 5.18 nm, corresponding to the dimension of the supercell along the zigzag direction. To characterize interlayer bond formation and rupture, we used an interatomic distance criterion of 1.8 Å for atoms residing in different layers. More details regarding the reactive sliding dynamics simulation setup are provided in SI Section S1.

We first examined the impact of external normal load on the bonding dynamics and sliding friction. Figure 1b displays four representative friction traces obtained at a temperature of *T* = 300 K and a sliding velocity of *v* = 10 m/s, under external normal loads of σ = 0, 1, 2, and 3 GPa. Similar to previous results,^34^ we find that under zero normal load, no apparent covalent bonding between the two layers occurs even when the defects are eclipsed, leading to a relatively smooth shear trace and low friction. Increasing the load to 1 or 2 GPa results in the formation of covalent bonds, as manifested by the pronounced shear-stress peak appearing at a lateral displacement of *x* ≈ 2.75 nm, slightly beyond the eclipsed defects configuration. At a higher normal load to 3 GPa, two additional shear-stress features appear (at *x* ≈ 2.55 nm and *x* ≈ 3.15 nm), corresponding to consecutive bond formation and rupture events (see SI Movies S1, S2, and S3 for single and multiple binding events). These results suggest that, beyond the increase of friction with normal load due to enhanced Pauli repulsions at the pristine interface regions, covalent bonding in defected regions introduces a significant positive contribution to the kinetic friction coefficient.

Along the chosen sliding path, we identified five distinct interlayer bonded pairs, involving the dangling atoms of the defects. To characterize those, we mark the three dangling atoms in top layer as T_1_, T_2_, and T_3_, and corresponding atoms in bottom layer as B_1_, B_2_, and B_3_. Figure 1c shows a representative trajectory obtained under an external normal load of 3 GPa that incorporates three regions for consecutive bond formation and rupture events. The first event (“region 1”) occurs at lateral displacement range of 2.40 nm < *x* < *x*_1_^max^ = 2.60 nm featuring binding between the top rightmost (T_2_) and the bottom leftmost (B_1_) dangling atoms, which we mark as T_2_B_1_. The second event (“region 2”) occurs at a lateral displacement range of 2.60 nm < *x* < *x*_2_^max^ = 2.85 nm, featuring a T_2_B_2_ covalent bond. We note that at this eclipsed configuration, the probabilities of forming T_1_B_1_ or T_3_B_3_ bonds are comparable to that of the featured T_2_B_2_ bond, and the identity of the bond formed is determined stochastically. We further note that we did not identify any events of simultaneous multiple interlayer bond formation in this region over 100 independent trajectories. We attribute this to the fact that the simultaneous formation of two or three interlayer bonds in this region involves a significantly higher transition energy barrier (TEB) as compared to the single bond formation (see SI Section S2). Finally, the third binding event (“region 3”) occurs at a lateral displacement range of 2.85 nm < *x* < *x*_3_^max^ = 3.20 nm, demonstrating a T_1_B_2_ bonding configuration.

Bond formation in reactive sliding interfaces is a stochastic process, influenced by the external normal load, sliding velocity, and temperature, as validated through MD simulations (see Figure 2 and SI Section S5). In the top panels of Figure 2 and SI Figure S4, we present the total probabilities associated with the three distinct regions outlined in Figure 1c (marked as *p*_1_, *p*_2_, and *p*_3_), whereas in the bottom panels of the figure we show the individual bond formation probabilities of the three different pairs in region 2 (marked as *p*_T_1_B_1__, *p*_T_2_B_2__, and *p*_T_3_B_3__). All bond formation probabilities are found to grow with increasing normal load (see Figure 2a and SI Figure S4a), leading to the enhancement of the shear stress peak (see Figure 1b). At a sliding velocity of *v* = 10 m/s, *p*_2_ is much larger than *p*_1_ and *p*_3_ for all normal load considered, reaching 100% at 2.5 GPa. Specifically, in region 2, T_2_B_2_ and T_3_B_3_ pairs are more likely to bind than T_1_B_1_ with saturation probabilities of *p*_(T_2_B_2_)_ ≈ *p*_(T_3_B_3_)_ ≈ 2*p*_(T_1_B_1_)_ ≈ 40%. Interlayer bond formation probability increases with temperature (see Figure 2b). In region 2, characterized by a lower TEB (see SI Figure S2a), it saturates around room temperature, whereas in regions 1 and 3 a higher saturation temperature is observed.

**Figure 2:**
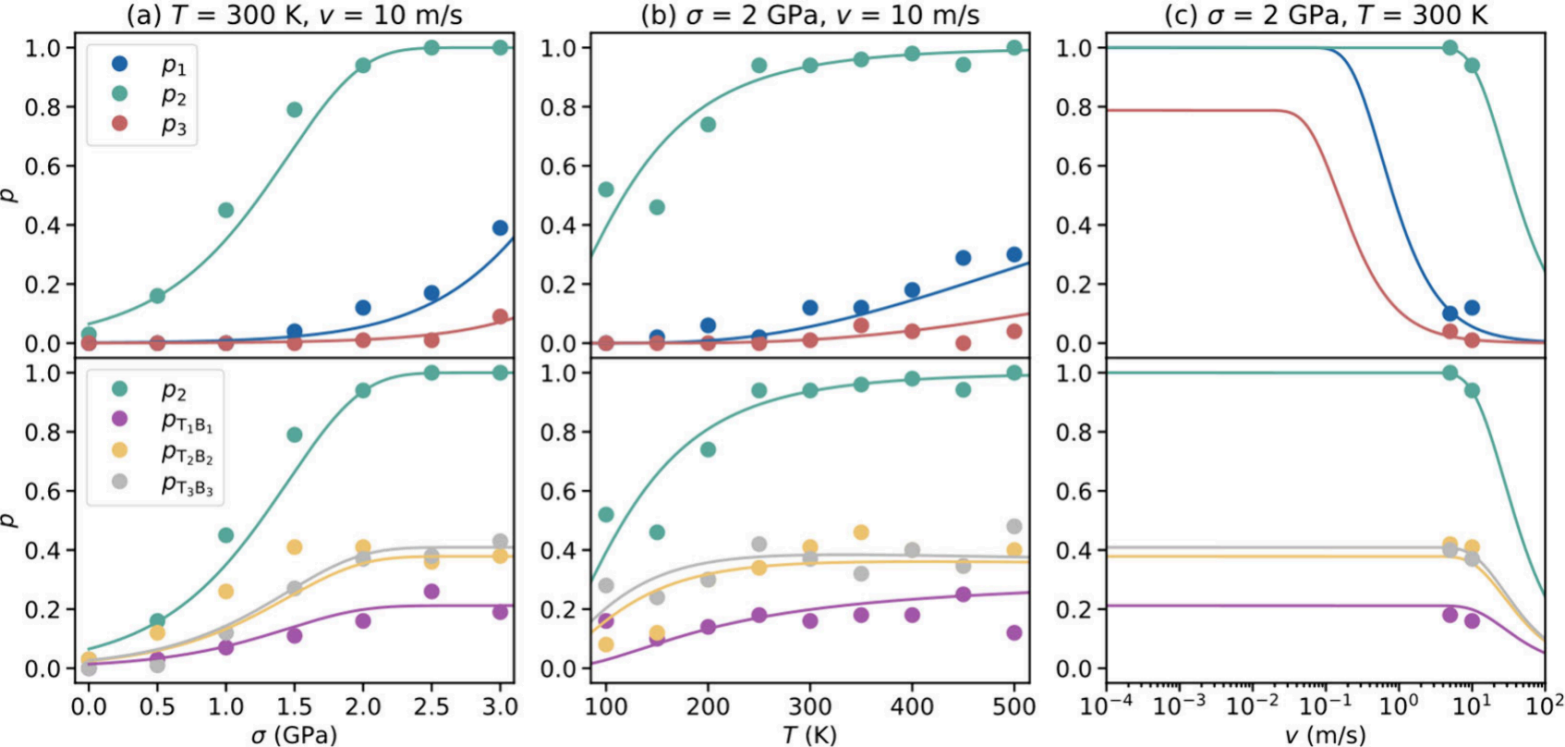
Stochastic interlayer binding dynamics. Interlayer bond formation probability of various atom pairs in a 9.43° twisted V_1_V_1_ bilayer as a function of (a) external normal load, (b) temperature, and (c) sliding velocity, obtained from ensemble MD simulations (circles) and the kinetic model (eqs 4–7) (solid lines). In the top panels, *p*_1_, *p*_2_, and *p*_3_ denote probabilities for the three distinct binding regions. The bottom panels present the overall probability for region 2 and the corresponding contribution of each specific binding atomic pair.

The atomistic simulations presented above provide a microscopic understanding of the chemifriction mechanisms involving interlayer bond formation and rupture. Nonetheless, they are limited in their ability to reproduce experimentally relevant conditions, such as low sliding velocities (see Figure 2c) and long sliding scenarios. To access such parameter regimes, we developed a physically motivated phenomenological kinetic model, derived based on the twisted V_1_V_1_ bilayer simulation results. The model describes the chemifriction component in terms of the rate of shear-induced interlayer bond formation and its survival probability. The former is given by the Arrhenius law:^43−45^
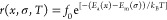
1where *f*_0_ is the attempt frequency, *k*_B_ is the Boltzmann constant, *E*_a_(*x*) is the TEB in the absence of external load, and *E*_m_(σ) is the effective TEB reduction due to the application of normal pressure, σ. In our model, *E*_a_(*x*) is assumed to have a parabolic dependence on the lateral defect displacement (*x*), obtaining a minimum (*E*_min_) at the eclipsed configuration (corresponding to a displacement of *x*_e_): *E*_a_(*x*) = *E*_min_ + β(*x* – *x*_e_)^2^, where β is a positive constant. Furthermore, the TEB reduction is assumed to be linear with normal pressure *E*_m_(σ) = *ασ*,^46−48^ where the activation volume, α, serves here as a measure of the response of chemical reactivity to external load.^43^

To describe the bond formation kinetics, we introduce the probability of the system to avoid interlayer bonding, *s*(*x*,σ,*v*,*T*), up to sliding displacement, *x*, at a sliding velocity, *v*, which is assumed to satisfy an irreversible first-order rate equation:
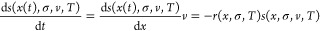
2Substituting eq 1 into eq 2 and solving for *s*(*x*,σ,*v*,*T*), we obtain
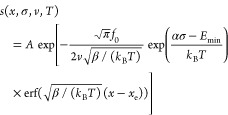
3where  is the Gaussian error function^49^ and *A* is a normalization constant, set to satisfy the initial condition *s*(*x*=0) = 1, where *x* = 0 is the interlayer shift at which the lateral distance between the two defects is the largest within our laterally periodic supercell (see Figure 1a). In particular, for region 1, where only a single binding scenario is possible, eq 3 yields a bond formation probability of
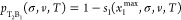
4where *s*_1_ is given by eq 3 with *E*_min_ = *E*_min_^(T_2_B_1_)^ being the load-free energy barrier for T_2_ B_1_ bond formation in this region.

For region 2, where three potential binding scenarios exist, the rate term of eq 1 is written as a sum of the three possible bond formation rates. This, in turn, yields a total bond formation probability of (see SI Section S3 for detailed derivations):
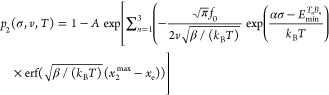
5where *E*_min_^(T_*n*_B_*n*_)^ is the barrier height associated with the load-free formation of a T_*n*_B_*n*_ bond, with *n* = 1, 2, or 3. Using this expression one can approximate the formation probability of individual interlayer pairs via
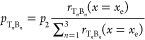
6where the bond formation transition rate is measured at the eclipsed configuration, *x*_e_, where it is maximal.

In region 3, the binding probability depends on the sliding history. If in region 2 the bond T_1_B_1_ is formed (and eventually ruptures), healing consistently occurs by a transfer of an atom between the layers, yielding a transition from a V_1_V_1_ to a V_0_V_2_ configuration (pristine bottom layer and a double vacancy in the top layer). In such a case, interlayer bonding in region 3 is prevented. Hence, the probability for bond formation in this region is given by
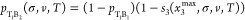
7where *s*_3_ is given by eq 3 with *E*_min_ = *E*_min_^(T_1_B_2_)^ being the load-free energy barrier for T_1_B_1_ bond formation in this region.

In our calculations we chose *f*_0_ = 100 GHz,^50^ and set *x*_e_ to be 1.52, 1.73, and 1.97 nm, corresponding to the eclipsed dangling atoms configurations in each region, respectively. The values of the rest of the kinetic model parameters are fitted to obtain good agreement with our atomistic simulation results (see Figure 2 and SI Figure S4). The fitted values are α = 0.048 eV/GPa, β = 2.5 eV/nm^2^, and *E*_min_ = 0.185, 0.125, 0.110, 0.108, and 0.220 eV, for the T_2_B_1_, T_1_B_1_, T_2_B_2_, T_3_B_3_, and T_1_B_2_ bond pairs, respectively. The value of α corresponds well to values estimated for similar reaction processes studied in AFM experiments, such as the wear of amorphous hydrogenated carbon^51^ and crystalline Si tips^52^ on diamond (see Table 1 of ref (43)). The values of *E*_min_ agree well with those predicted by nudged elastic band (NEB) calculations (see SI Table S1). The chosen values for α and β were further validated through zero temperature reactive sliding dynamics as detailed in SI Section S4.

One can now use the developed phenomenological model to evaluate the interlayer bonding probability at experimentally relevant sliding velocities. As can be seen in Figure 2c (solid lines) and SI Figure S4c, at the low sliding velocity regime the binding probability saturates in all three regions. Specifically, under a normal load of 2 GPa, *p*_1_ and *p*_2_ reach the maximal value of 1 below sliding velocities of ∼0.1 m/s and ∼5 m/s, respectively, whereas *p*_3_ saturates at 0.8 below a sliding velocity of ∼0.01 m/s. We note that the latter does not reach the maximal value of 1 due to the healing process discussed above, suggesting that when a T_1_B_1_ pair forms in region 2, binding in region 3 is inhibited. As shown in SI Figure S5, our model predicts that the saturation velocity decreases exponentially with reduction of the normal load.

Since friction in our system is dominated by interlayer chemical bonding, one can use the bonding probability functions derived above to estimate the dependence of the frictional shear stress on external parameters, such as the normal load and the sliding velocity. Marking by *f*_T_*i*_B_*j*__ (*x*) the shear stress trace associated with the T_*i*_B_*j*_ bonding event (*i* and *j* representing dangling atom indices in the top and bottom layers, respectively), the overall shear stress trace can be written as the following sum:
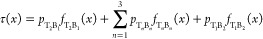
8Here, based on the atomistic simulation results, we assume that binding events in different regions are spatially separated and that in region 2 only a single binding event per trace can occur. The individual shear stress traces can be approximated using harmonic springs of effective stiffness, *k*_eff_^T_*i*_B_*j*_^, spanning from the initial bond formation position, *x*_b_^T_*i*_B_*j*_^, to the rupture position, *x*_b_^T_*i*_B_*j*_^ + *l*_r_^T_*i*_B_*j*_^(*T*,*v*), yielding
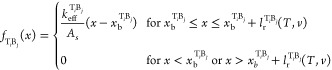
9where *A*_s_ is the in-plane surface area. Here, the bond formation position and spring stiffnesses are assumed to be constant, whereas the bond rupture lengths, *l*_r_(*T*,*v*), are assumed to depend only on the sliding velocity and the temperature.^53−57^*l*_r_ can be evaluated by the surface area encompassed by the bond survival probability distribution across the sliding displacement. This probability can be estimated via the same kinetic model used above (eqs 1 and 2) for bond formation, where the bond rupture energy barrier in the finite harmonic potential well decreases quadratically with lateral interlayer displacement:
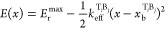
10and −*E*_r_^max^ is the harmonic potential minimum. Substituting eq 10 in eq 1 and solving for eq 2, one obtains the bond survival probability:^58,59^
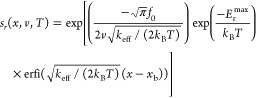
11where  is the imaginary error function. This allows us to evaluate the probability density function for bond rupture at a displacement interval between *x* and *x* + d*x* as . Averaging the bond rupture length over this probability density function yields the mean bond rupture length:
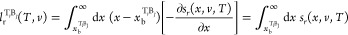
12where we have used integration by parts and assumed that *s*_r_(*x*,*v*,*T*) vanishes at *x* → ∞. This allows us to evaluate the shear stress traces, *f*_T_*i*_B_*j*__(*x*), of eq 9 with the parameters extracted from the reactive sliding dynamics simulations (see SI Section S6 for details), which together with eqs 4–7 for the bond formation probabilities, *p*_T_*i*_B_*j*__(σ,*v*,*T*), provide an estimation of the overall frictional shear stress traces via eq 8.

The derived phenomenological model reproduces the shear stress traces obtained from the fully atomistic simulation in a broad range of normal loads with remarkable success (see Figure 1b). Given the reliability of the model and the fact that all its assumptions and parameters should hold also outside the conditions characterizing our simulation results, we may now use it to predict chemifrictional effects in experimentally accessible sliding velocities.^10,39^Figure 3 shows the mean kinetic frictional stress dependence on the normal load (Figure 3a) and sliding velocity (Figure 3b) under such conditions. As expected, at the high-velocity regime there is a monotonic increase of friction with normal load, due to the corresponding increase of bond formation probability (see Figure 2a and SI Figure S4a). Notably, at low velocities the friction is found to be nearly independent of the applied load. This is attributed to the fact that the bond formation probability itself becomes independent of normal load at this experimentally relevant regime (see SI Figure S6). For the velocity dependence of friction, we find two distinct regimes. At experimentally relevant velocities friction increases logarithmically. This can be rationalized by the fact that the bond rupture length increases with sliding velocity,^58,59^ because thermal fluctuations do not have sufficient time to assist the bond rupture barrier crossing. A turnover is predicted to occur at higher velocities, where friction is found to drastically decrease with sliding velocity, due to reduced probability of interlayer bond formation. Notably, at the high velocity regime, both the load and the velocity dependence of the frictional stress exhibit a wavy structure. This is attributed to the difference in the TEBs associated with various interlayer bonding pairs that are activated at different velocities and normal loads.

**Figure 3:**
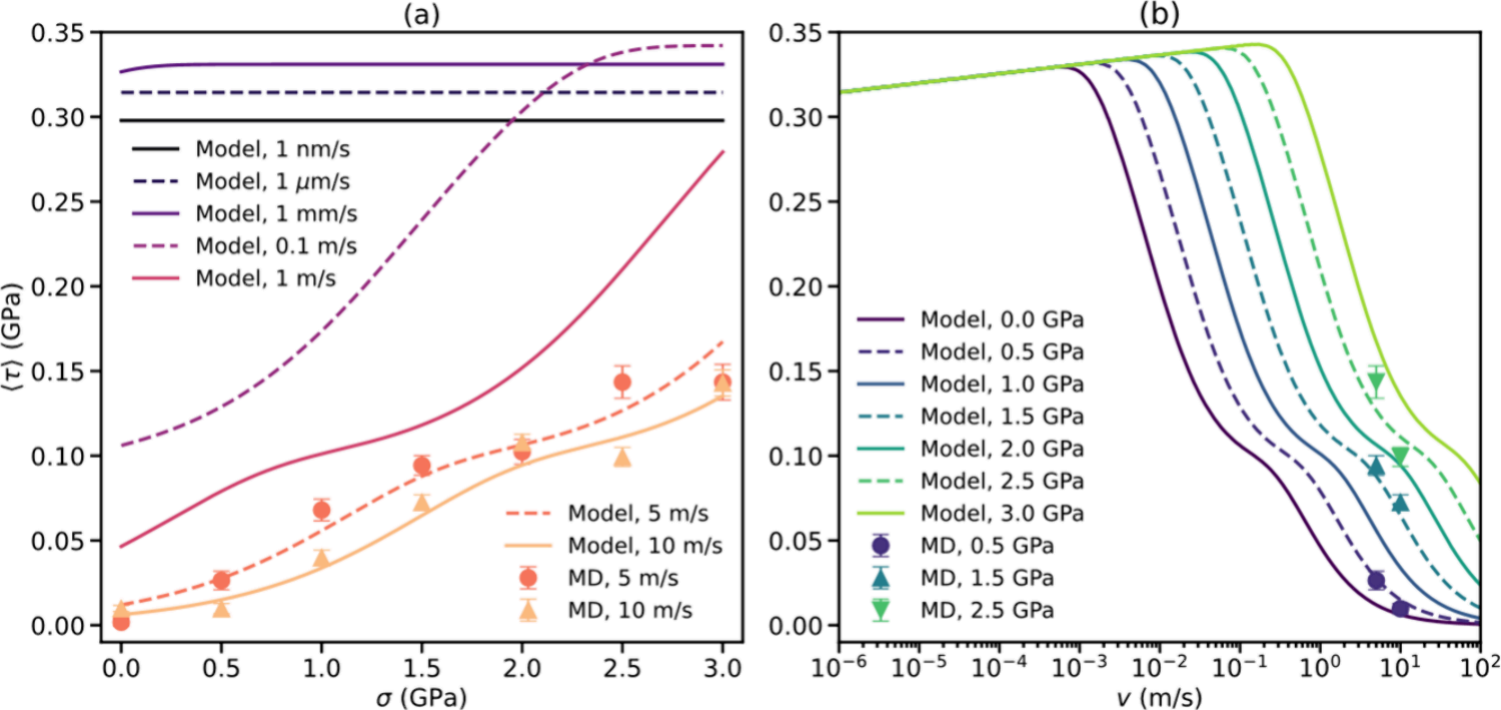
Normal load (a) and velocity (b) dependence of the chemical contribution to the kinetic frictional stress of a twisted V_1_V_1_ bilayer at a temperature of 300 K. Symbols represent atomistic simulation results, and lines represent model predictions (eq 8). Error bars for MD results are calculated from the standard error of the mean across ensembles of 100 trajectories for 10 m/s sliding velocity results and 50 trajectories for 5 m/s results. To isolate the chemifriction effect, we subtract from the atomistic simulation results the (nearly constant) frictional stress obtained for the pristine V_0_V_0_ bilayer (∼0.008 GPa; see Figure 5c of ref (34)), which is not considered by our phenomenological model.

As mentioned earlier, a unique sliding induced healing process occurs when the T_1_B_1_ pair bond ruptures, involving the transfer of the T_1_ atom from the top layer to the bottom layer. This results in a V_0_V_2_ configuration, where the bottom layer is pristine, and the top layer includes a double vacancy (see the inset of Figure 4a and SI Movie S4). NEB calculations reveal that the TEB for this healing transition (0.67 eV) is significantly lower than that for reverting back to the original V_1_V_1_ configuration (1.74 eV) upon bond rupture (see SI Section S2 for computational details). The resulting V_0_V_2_ configuration presents smooth superlubric motion, similar to that of the pristine V_0_V_0_ bilayer for all normal loads considered (see Figure 4b). At a temperature of *T* = 300 K and a sliding velocity of *v* = 10 m/s, the coefficient of friction of the V_0_V_2_ configuration is 3.2 × 10^–4^, compared to 3.8 × 10^–4^ for the pristine bilayer and 6.3 × 10^–3^ for the V_1_V_1_ configuration, as shown in Figure 4c. Since the healing process is initiated above a normal load of ∼0.5 GPa, we expect a reduction of friction with increasing normal load around this threshold, resulting in a negative differential friction coefficient.

**Figure 4:**
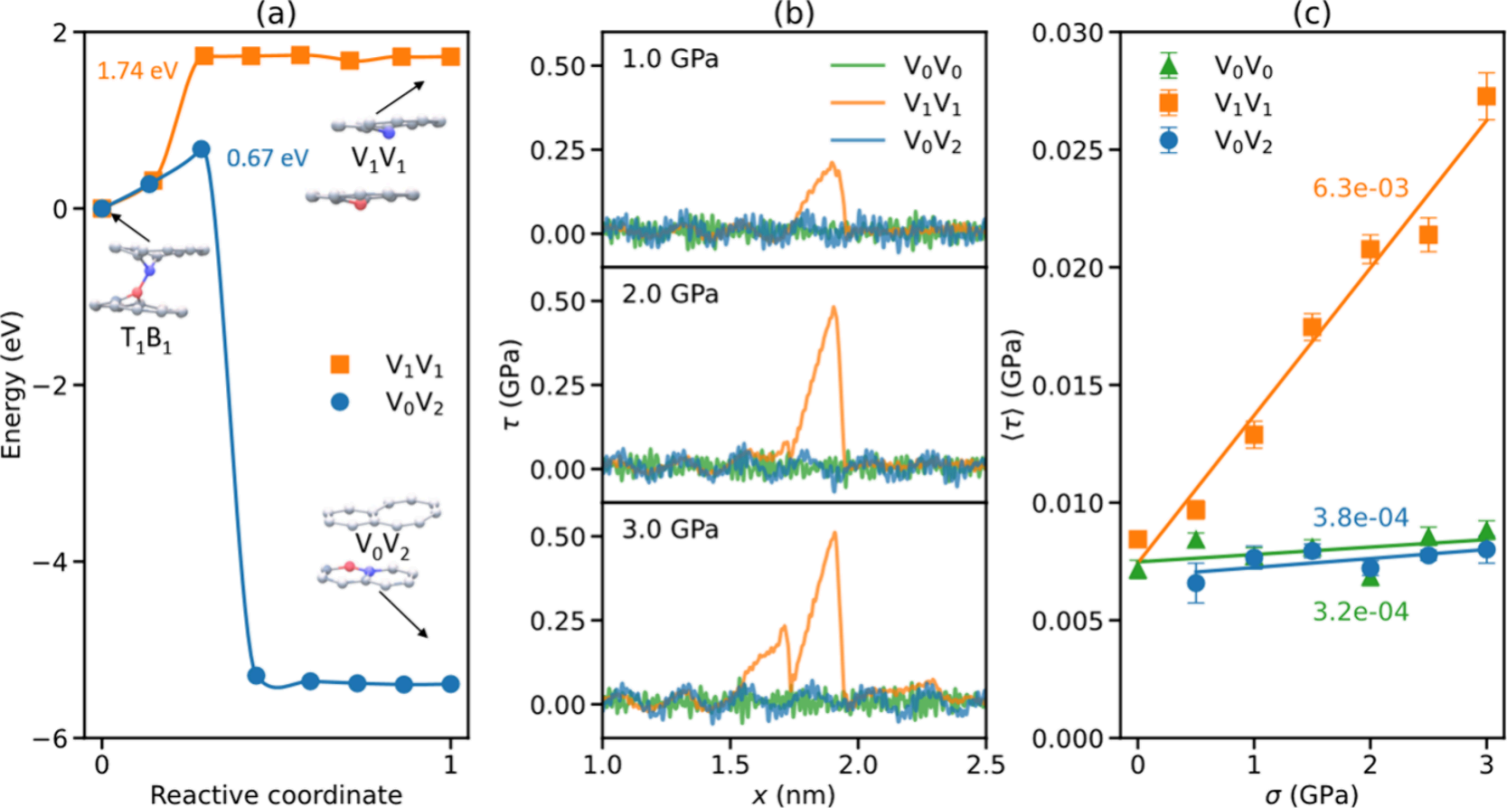
Sliding induced healing. (a) NEB results, obtained under zero normal load, for two reaction pathways of T_1_B_1_ bond rupture, one leading to the V_1_V_1_ configuration (orange) and the other to the V_0_V_2_ configuration (blue). Symbols and solid lines represent the energies of individual images and a cubic polynomial interpolation, respectively. (b) Frictional stress traces of the V_0_V_0_ (green), V_1_V_1_ (orange), and V_0_V_2_ (blue) configurations under normal loads of 1 (top subpanel), 2 (middle subpanel), and 3 GPa (bottom subpanel). (c) Average frictional stress calculated for the three configurations as a function of normal load obtained from traces such as those presented in panel b.

The revealed sliding-induced healing process can serve to constitute a run-in procedure, where presliding cycles reduce friction. To demonstrate this, we conducted repeated forward and backward sliding simulations for the twisted V_1_V_1_ bilayer at *T* = 300 K and *v* = 10 m/s. To accelerate the simulations, the maximal lateral distance between two vacancies was set at 0.74 nm, corresponding to a separation of three intralayer lattice vectors. Each trajectory consisted of 20 cycles, with a forward and backward sliding distance of 1.5 nm each. Kinetic frictional stress results were averaged over 30 independent trajectories for σ = 0.5 GPa, and 10 trajectories for σ = 1.0 and 2.0 GPa. At a normal load of σ = 1.0 GPa and above, we find a sharp drop in the corrugation of the average friction stress traces after a few cycles (<5; see Figure 5a), corresponding to a transition from the V_1_V_1_ to the V_0_V_2_ configuration (manifested as a reduction in the survival probability of the V_1_V_1_ configuration and overall trace averaged frictional stress, see Figure 5b). Notably, Figure 5b also shows that the healing transition is accelerated under higher normal loads, due to the corresponding increase in the T_1_B_1_ pair interlayer bond formation probability in region 2 (see Figure 2a). These results thus demonstrate that the suggested run-in process leads to a healing-induced transition to a superlubric state, reminiscent of that of the pristine interface, which can be accelerated by increasing the normal load.

**Figure 5:**
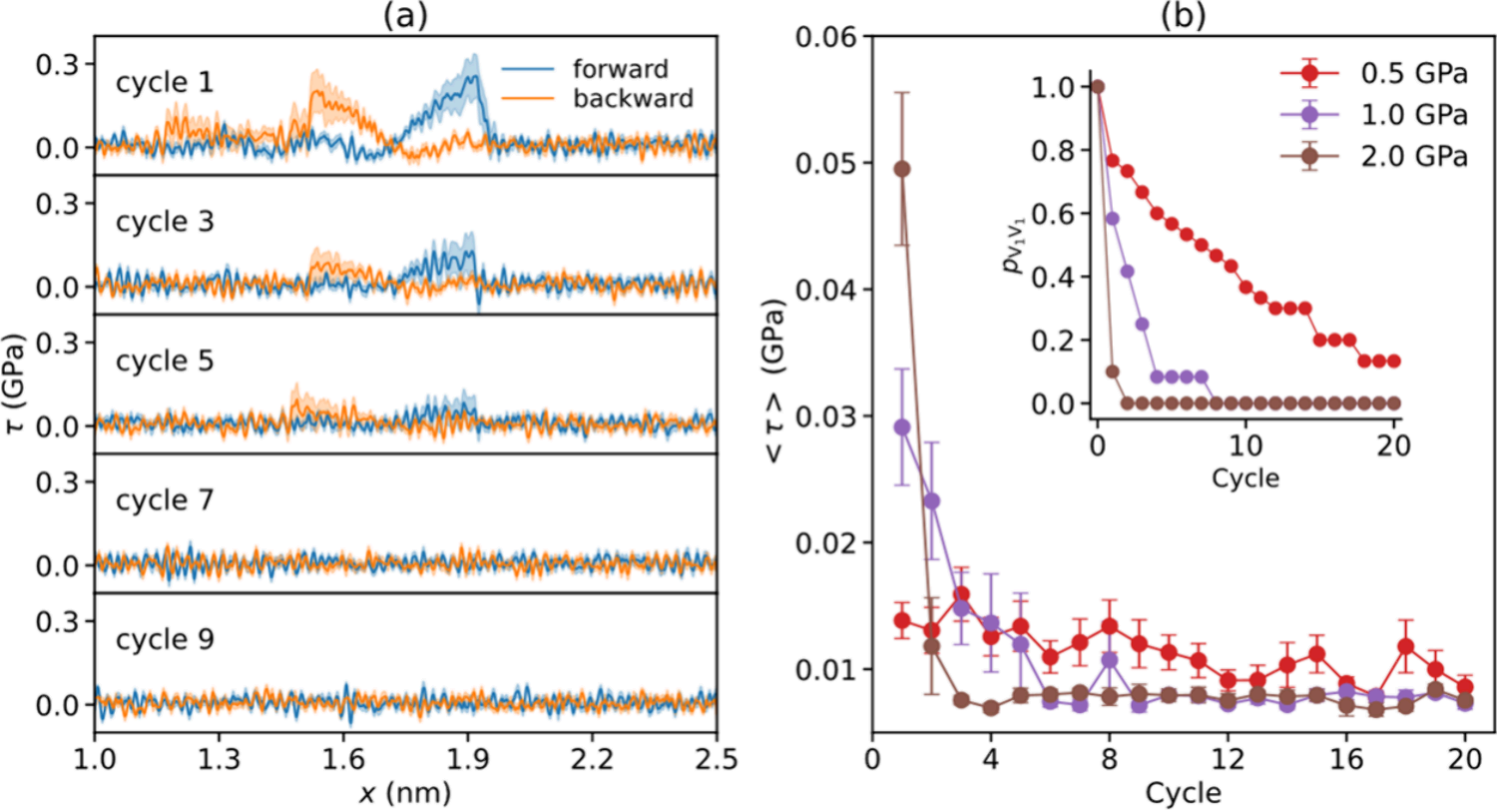
Simulations of the run-in procedure. (a) Averaged friction stress traces (blue and orange lines represent the trace and retrace, respectively) obtained for nine sliding simulation cycles of a twisted bilayer starting in a V_1_V_1_ configuration under an external normal load of 1.0 GPa. Here, averaging is performed over 10 independent trajectories. The shaded regions mark the standard error of the mean. (b) Evolution of the frictional stress (main panel, averaged over the entire average trajectory) and the survival probability of the V_1_V_1_ configuration (inset, *p*_V_1_ V_1__) as a function of cycle number, calculated under external normal loads of 0.5 (red), 1.0 (purple), and 2.0 (brown) GPa.

In summary, using a recently developed machine learning potential trained against ab initio reference data, we conducted reactive sliding dynamics simulations of a twisted incommensurate defected bilayer graphene interface. Our results reveal a unique microscopic interlayer bonding mechanism underlying *chemifriction* that may *impede* superlubric sliding. This mechanism involves consecutive stochastic bond formation and rupture events, that are spatially separated but not necessarily independent. Notably, an intricate healing process, involving interlayer atomic transfer, was identified that leads to the occurrence of negative differential friction coefficients and may be harnessed to design a friction reduction run-in procedure. Leveraging the derived atomistic insights, we developed a physically motivated phenomenological model that illustrates the interplay between bond formation kinetics, which are predominantly activated at high sliding velocities, and rupture kinetics, which become significant at lower velocities. This model allows us to predict chemifrictional effects in the experimentally accessible low sliding velocity regime, revealing a distinct transition from a logarithmic increase to a logarithmic decrease of frictional stress with velocity. While one may naively assume that chemifriction would always increase friction, our results demonstrate that under appropriate conditions, it may actually lead to the reduction of friction and *support* superlubric sliding. The frictional mechanism discovered herein based on unpassivated single vacancy defect models may serve as a basis for understanding chemifrictional behavior under more complex scenarios involving different types of defects, chemical passivation, and intercalants.

## Supporting Information Available

Reactive sliding dynamics simulation setup; nudged elastic band calculations; bond formation probability in region 2; reactive sliding dynamics at zero temperature; additional reactive sliding dynamics simulations at finite temperature; parameters for estimating bond rupture length (PDF)
Movie S1 showing single binding event (AVI)
Movie S2 showing two consecutive binding events (AVI)
Movie S3 showing three consecutive binding events (AVI)
Movie S4 showing V_0_V_2_ configuration with pristine bottom layer and double vacancy in top layer (AVI)
Transparent Peer Review report available (PDF)

## Supplementary Material

jz5c00193_si_001.pdf

jz5c00193_si_002.avi

jz5c00193_si_003.avi

jz5c00193_si_004.avi

jz5c00193_si_005.avi
