# Supplementary material for: Chemifriction and Superlubricity: Friends or Foes?
Source: J Phys Chem Lett. 2025 Mar 13;16(11):2934–41. doi: 10.1021/acs.jpclett.5c00193 (PMC11931540; doi:10.1021/acs.jpclett.5c00193)
Supplement: Supplementary file 1 — jz5c00193_si_001.pdf [file jz5c00193_si_001.pdf]

Supplemental Material for **"Chemifriction and Superlubricity: Friends or Foes?"**

Penghua Ying[1], Xiang Gao[1], Amir Natan,[2] Michael Urbakh[1], Oded Hod[1]

[1] *Department of Physical Chemistry, School of Chemistry, The Raymond and Beverly Sackler Faculty of Exact Sciences and The Sackler Center for Computational Molecular and Materials Science, Tel Aviv University, Tel Aviv 6997801, Israel*

[2] *Department of Physical Electronics, Tel-Aviv University, Tel-Aviv, 6997801, Israel*

This supporting information document includes the following sections:

1. Reactive sliding dynamics simulation setup
2. Nudged elastic band calculations
3. Bond formation probability in region 2
4. Reactive sliding dynamics at zero temperature
5. Additional reactive sliding dynamics simulations at finite temperature
6. Parameters for estimating bond rupture length

1. Reactive sliding dynamics simulation setup

The twisted V_1V_1 bilayer model system was constructed following the method described in Muniz and Maroudas, 2012 (see Fig. 1 of the main text). Periodic boundary conditions were employed in the in-plane directions with lateral dimensions of $5.18 \times 1.50\ \mathrm{nm}^2$, and a vacuum layer of ~ 15 Å was used in the out-of-plane direction. These supercell dimensions were found to be sufficiently large to avoid interactions between adjacent defect images.

During the reactive sliding simulations the top layer was laterally driven along the zigzag (x-axis) graphene lattice direction by a rigid slider (modeled by a rigid pristine graphene layer) with a spring of stiffness $K_1 = 50\ \mathrm{N/m}$, and the bottom layer was anchored with harmonic springs of the same stiffness, $K_2 = 50\ \mathrm{N/m}$, to their original positions, effectively simulating a double-layer substrate (see Fig. S1 and SM Section 4 of Ying et al., 2024). In each trajectory, unless otherwise specified, a total relative sliding displacement of $5.18\ \mathrm{nm}$ was implemented. The instantaneous frictional stress trace was calculated as $\tau = K_1(x - X_{com})/A_s$, where X_{com} is the center of mass x coordinate of the top layer, $x = vt$ is the displacement of the rigid layer, and A_s is the contact area, defined as the interface area of the V_1V_1 bilayer supercell. For each trajectory, the kinetic friction was then determined as the mean shear stress $\langle\tau\rangle$ over the entire trace. To evaluate the bond formation and rupture probabilities we captured 518 atomic snapshots, corresponding to displacement intervals of 0.1 Å. For each snapshot, the binding state of each dangling pair was determined as bonded or non-bonded based on a cutoff distance of 1.8 Å.

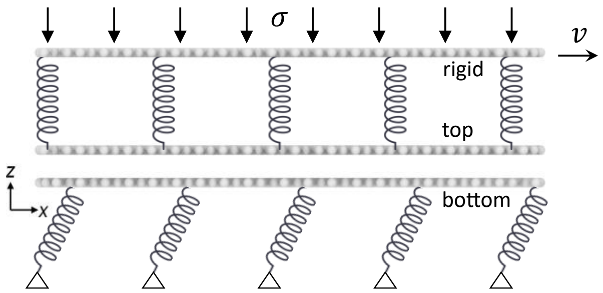

Fig. S1: A schematic representation of the simulation setup.

We consider the influences of sliding velocity, v, external normal load, σ, and temperature, T, on the sliding friction of the reactive sliding interfaces. The sliding velocity was imposed by moving the rigid slider at a target constant value; the external normal pressures was applied by exerting a vertical force to each atom of the rigid slider (see Fig. S1); and temperature was set by using a Langevin thermostat (Schneider and Stoll, 1978) applied to all sliding interface flexible atoms in all three direction with a damping parameter of $1\ \mathrm{ps}$. Given the stochastic nature of interlayer bonding kinetics, we considered 100 independent sliding trajectories (each with a different random seed of the initial atomic velocity distribution) to calculate the average friction trace and corresponding kinetic friction for the V_1V_1 bilayer at $T = 300\ \mathrm{K}$ and $v = 10\ \mathrm{m/s}$; and 50 trajectories in all other cases investigated. The kinetic friction stress was evaluated from the average of these independent trajectories, and the standard error was calculated to obtain error bars.

2. Nudged elastic band calculations

To evaluate the transition energy barrier (TEB) for interlayer bond formation of different atomic pairs, we used the nudged elastic band (NEB) method (Jónsson et al., 1998), as implemented in the Atomic Simulation Environment (ASE) package (Larsen et al., 2017). Atomic forces were calculated using ASE, interfaced with the developed NequIP model (available at https://doi.org/10.5281/zenodo.11473281). Atomic position optimizations for the initial (reactants) and final (products) structures were performed using the FIRE algorithm (Bitzek et al., 2006) with a fixed supercell box and an atomic force convergence criterion of $0.01\ \mathrm{eV/Å}$. Eighteen interior images were interpolated between the initial and final optimized structures to establish a preliminary reaction path. All replicas were connected by springs of stiffness of $0.1\ \mathrm{eV/Å^2}$ and the energy of the entire band was minimized using the FIRE algorithm with a force tolerance of $0.02\ \mathrm{eV/Å}$ to determine the reaction path and the TEB values (see Fig. S2).

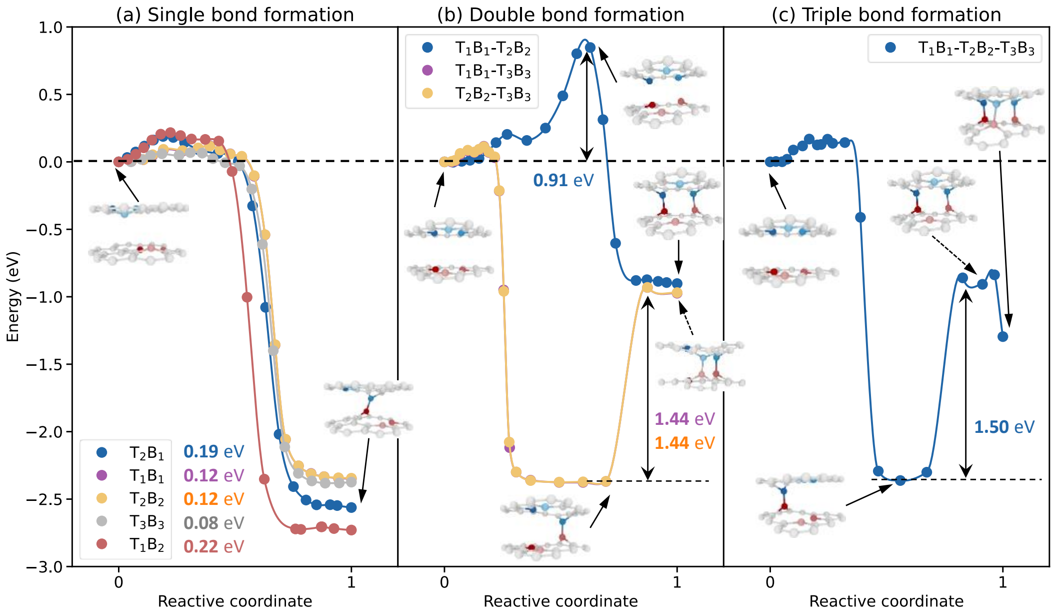

Fig. S2. NEB predictions of the reaction paths and TEBs for different interlayer bond formation scenarios: (a) single bond formation of five different atomic pairs across the three displacement regions; (b) simultaneous double bond formation in region 2 for three different atomic pairs; and (c) simultaneous triple bond formation in region 2. The insets in (a-c) are representative snapshots along the reaction path.

Figure S2a displays the reaction paths for single interlayer bond formation of five different atomic pairs (see Fig. 1c of the main text). The $\mathrm{T_2B_1}$ (region 1) and $\mathrm{T_1B_2}$ (region 3) bond formation energy barriers (estimated as the energy difference between the saddle-point and the unbound state) of 0.19 and $0.22\ \mathrm{eV}$, respectively, are found to be considerably larger than the corresponding barriers $(\sim 0.1\ \mathrm{eV})$ for the other three pairs, i.e., $\mathrm{T_1B_1}$, $\mathrm{T_2B_2}$, and $\mathrm{T_3B_3}$ in region 2. As can be seen in Table S1, the interlayer bond formation TEB values obtained using the atomistic simulations are well reproduced by the kinetic model. The bond rupture TEBs (estimated as the energy difference between the saddle-point and the bonded state) are significantly larger, lying in the range of $2.5 - 3.0\ \mathrm{eV}$, which is on the order of the values extracted from MD simulations (see Table S2).

As mentioned in the main text, in region 2 we observed that only a single covalent interlayer bond forms throughout all MD trajectories. This phenomenon is attributed to the significantly higher TEBs associated with the simultaneous formation of double or triple bonds, as demonstrated in Figs. S2b and S2c. For instance, the consecutive formation of $\mathrm{T_1B_1}$ and $\mathrm{T_2B_2}$ interlayer bonds involves a TEB of 0.91 eV, whereas for the sequential formations of the $\mathrm{T_1B_1}$ and $\mathrm{T_3B_3}$ bond pair and of the $\mathrm{T_2B_2}$ and $\mathrm{T_3B_3}$ bond pair, the second bond formation is associated with a TEB of 1.44 eV (see Fig. S2b). Similarly, the formation of three bonds involves a TEB of 1.50 eV for the second binding event. Notably, even in this case, forming the third bond requires overcoming a much lower TEB, $\sim 0.1\ \mathrm{eV}$ (see Fig. S2c), exemplifying that the formation of the second bond poses the primary challenge in the eventual formation of two or three interlayer bonds.

Table S1. TEBs for interlayer single bond formation involving five different atomic pairs, as predicted by NEB calculations and by the kinetic model.

Method	TEBs (eV)				
	T_2B_1	T_1B_1	T_2B_2	T_3B_3	T_1B_2
NEB	0.190	0.121	0.118	0.073	0.218
kinetic model	0.185	0.125	0.110	0.108	0.220

3. Bond formation probability in region 2

As noted in the main text, interlayer bond formation kinetics in region 2 involves three different atomic pairs: $\mathrm{T_1B_1}$, $\mathrm{T_2B_2}$, and $\mathrm{T_3B_3}$. Assuming that the three binding events are independent, the kinetic equation for the probability to be in the unbound state (Eq. 2 of the main text) is generalized as follows:

$$\frac{ds_2}{dt} = -s_2 \sum_{n=1}^{3} r_{\mathrm{T}_n\mathrm{B}_n}, \tag{S1}$$

where $r_{\mathrm{T}_n\mathrm{B}_n}$ is the formation rate of atomic pair $T_nB_n(n = 1, 2, 3)$. This additive form is valid as the probability of forming second and third bonds (simultaneously or in parallel) with the first one is extremely low according to our simulations (see also SM section 2 above).

Incorporating the expression for $r_{\mathrm{T}_n\mathrm{B}_n}$ from Eq. 1 in the main text into Eq. S1 yields:

$$\frac{ds_2}{dx} = \frac{-f_0 s_2}{v} \sum_{n=1}^{3} \exp\left(\frac{\alpha\sigma - \Delta E_{\mathrm{min}}^{\mathrm{T}_n\mathrm{B}_n} - \beta(x - x_e)^2}{k_B T}\right), \tag{S2}$$

where $\Delta E_{min}^{\mathrm{T}_n\mathrm{B}_n}$ denotes the corresponding load-free bond formation energy barrier. Solving this equation with the initial condition $s_2(x = 0) = 1$ (where the origin is assumed to be sufficiently far from the eclipsed configuration) yields:

$$s_2(x, \sigma, v, T) \;=\; A \cdot \mathrm{Exp}\left\{\sum_{n=1}^{3}\left[-\frac{\sqrt{\pi} f_0}{2v\sqrt{\beta/(k_\mathrm{B}T)}} e^{\left(\frac{\alpha\sigma - E_{\mathrm{min}}^{\mathrm{T}_n\mathrm{B}_n}}{k_\mathrm{B}T}\right)} \mathrm{erf}\left(\sqrt{\beta/(k_\mathrm{B}T)}(x - x_e)\right)\right]\right\} \tag{S3}$$

Following Eq. 4 of the main text, the bond formation probability in region 2, p_2, can be obtained from $p_2(\sigma, v, T) = 1 - s_2(x_{\mathrm{max}}, \sigma, v, T)$, yielding Eq. 5 of the main text.

4. Reactive sliding dynamics at zero temperature

Inspired by the two-state model introduced in Ref. (Gao et al., 2021), the zero temperature friction stress trace can be estimated as follows:

$$\tau = \frac{k_{\text{eff}}}{A_s}(x - x_b)\left[1 - H\left(E_b(\sigma, x)\right)\right]H\left(E_r(x)\right), \tag{S4}$$

where the bond formation TEB is given by $E_b(\sigma, x) = E_{min} + \beta(x - x_e)^2 - \alpha\sigma$, the bond rupture TEB reads as $E_r(x) = E_r^{max} - \frac{1}{2}k_{\text{eff}}(x - x_b)^2$, and A_s is the contact area. Here, the Heaviside step function, H, defines the threshold conditions for interlayer bond formation (when the corresponding barrier nullifies $E_b(\sigma, x) = 0$) and rupture (when $E_r(x) = 0$).

To validate Eq. S4, we conducted reactive sliding dynamics simulations for the twisted V_1V_1 bilayer at zero temperature ($T = 0$ K) and a sliding velocity of 10 m/s under different external normal pressures. Using the parameters appearing in Tables S1 and S2, Eq. S7 accurately reproduces the atomistic simulation results (see Fig. S3). Below 2.5 GPa, bond formation does not occur, leading to ultralow friction throughout the trajectory (see top sub-panels of Figs. S3a, b). Conversely, at or above 2.5 GPa, bond formation of the T_3B_3 pair is observed in region 2, generating a distinct peak around an interlayer displacement of 2.6 nm (see the lower subpanels of Figs. S3a, b), which is manifested as an abrupt increase of the kinetic friction (see Fig. S3c).

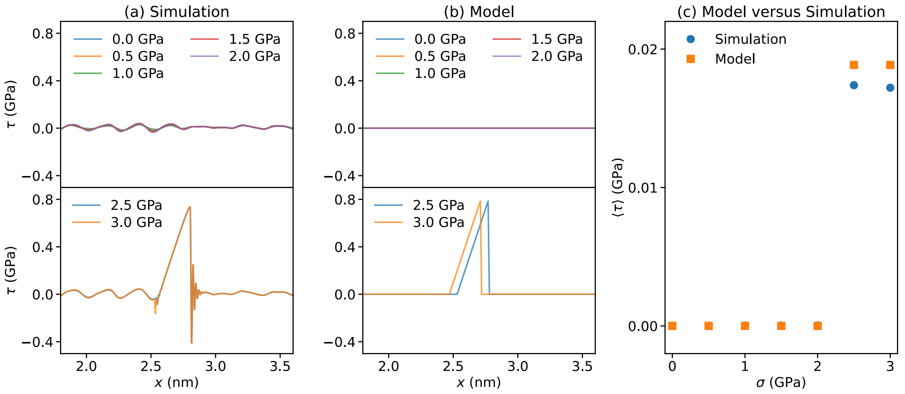

Fig. S3. Reactive sliding dynamics at zero temperature ($T = 0$ K). The friction trace of a twisted V_1V_1 bilayer under different external normal loads obtained from (a) atomistic MD simulation and (b) the model (Eq. S4). Panel (c) presents the kinetic friction (evaluated as the force average over the entire trace) as a function of normal load, comparing results from MD simulation (blue circles) with those derived from Eq. S4 (orange squares).

5. Additional reactive sliding dynamics simulations at finite temperature

Following the simulation results presented in Fig. 2 of main text, we conducted additional reactive sliding dynamics simulations at finite temperature to further validate our stochastic model for bond formation probability (Eqs. 4-7 of the main text). To that end, we performed simulations under varying external normal loads (see Fig. S4a), temperatures (see Fig. S4b), and sliding velocities (see Fig. S4c). Overall, the simulation results present the same qualitative picture as that seen in the main text and the kinetic model captures them well. Some quantitative variations are observed due to the different sliding conditions. For example, the transition pressure appearing in panel (a) downshifts with respect to that appearing in the main text, since the lower sliding velocity allows for more time for bond formation to occur.

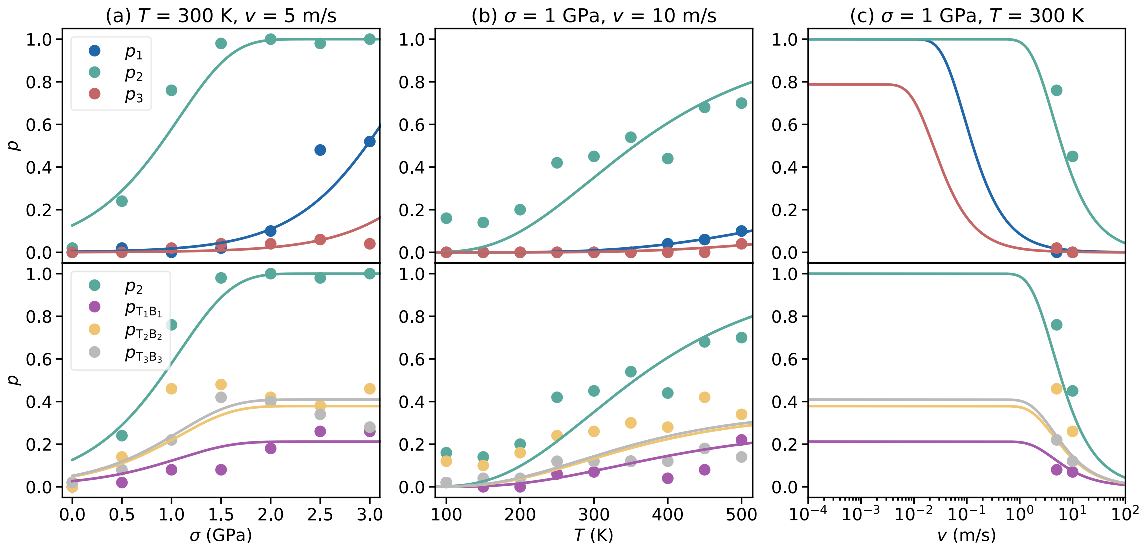

Fig. S4. Additional stochastic interlayer binding dynamics results. Interlayer bond formation probability of various atom pairs in a 9.43° twisted V_1V_1 bilayer as a function of (a) external normal load, (b) temperature, and (c) sliding velocity, obtained from ensemble MD simulations (circles) and the kinetic model (solid lines). The top panels show the probabilities for the three distinct binding regions, and the bottom panels present the overall probability for region 2 and the corresponding contribution of each specific binding atomic pair.

Similarly, the kinetic model predicts that the velocity, under which the bond formation probabilities saturate, downshifts with a reduction of the normal load (see Fig. S5a) and that the dependence is exponential (see Fig. S5b).

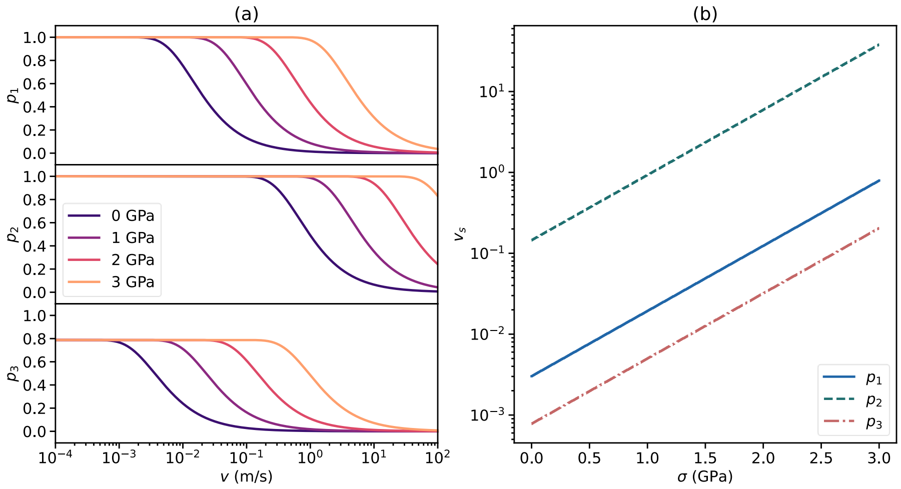

Fig. S5. (a) Interlayer bond formation probabilities as a function of sliding velocity, under various external normal loads, predicted by the kinetic model for regions 1 (p_1, top subpanel), 2 (p_2, middle subpanel), and 3 (p_3, lower subpanel). (b) Load dependence of the velocity under which the bond formation probability saturates, v_s, for the three sliding regions.

Finally, to explain the load-independent frictional stress observed in Fig. 3a of the main text, in Fig. S6 we use the kinetic model to plot the interlayer bond formation probabilities for the three regions as a function of normal load. Indeed, at experimentally relevant sliding velocities of $\lesssim 1\,\mathrm{mm/s}$, all interlayer bond formation probabilities, which dominate friction, are found to be independent of the normal load.

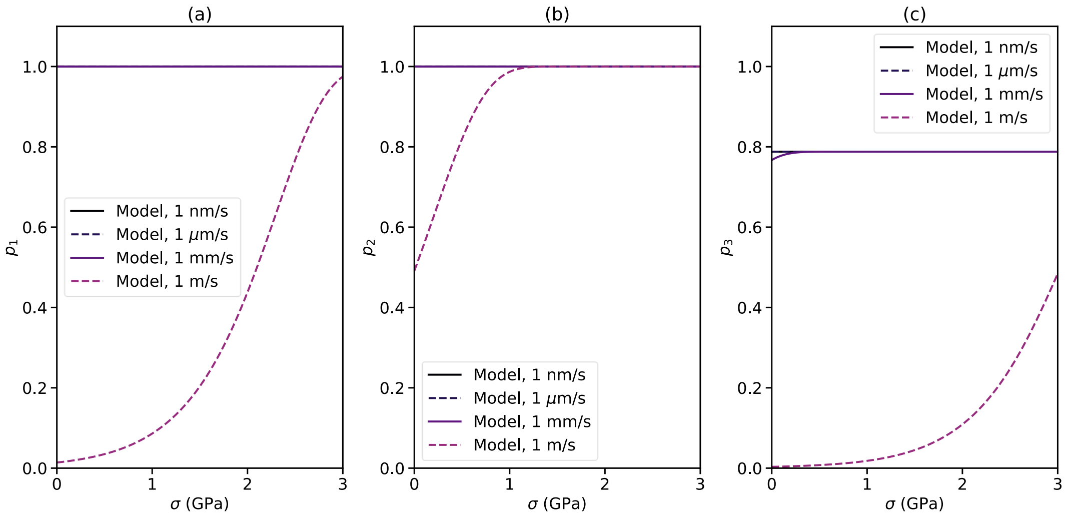

Fig. S6. Interlayer bond formation probability in (a) region 1, (b) region 2, and (c) region 3 for a 9.43° twisted V_1V_1 bilayer as a function of external normal load, as predicted by the kinetic model. For all three regions, four representative sliding velocities, i.e., $1\ \mathrm{nm/s}$, $1\ \mu\mathrm{m/s}$, $1\ \mathrm{mm/s}$, and $1\ \mathrm{m/s}$ are considered.

6. Parameters for estimating bond rupture length

In this section, we discuss the choice of parameters used for calculating the bond rupture length via Eq. 12 of the main text. In Fig. S7 we show the frictional stress traces for each bonded atomic pair obtained from reactive sliding dynamics simulations of the twisted V_1V_1 bilayer at a temperature of $T = 300$ K and a sliding velocity of $v = 10$ m/s, under three normal loads. For each atomic pair, we considered the friction trace section spanning the region between bond formation and rupture positions, as determined by observing snapshots along the trace. Based on these simulation traces, Fig. S8 shows the average bond formation position, x_b, bond rupture length, l_r, and effective bond stiffness, k_{eff} (obtained via linear fits of the traces appearing in Fig. S7), as functions of normal load. All three parameters for all pairs appear to be unaffected by the external normal load, in the considered range. Therefore, in Eq. 9 of the main text, x_b, l_r, and k_{eff} are taken to be load independent. The bond rupture TEB can then be estimated using the spring energy equation, $E_r^{\max} = \frac{1}{2} k_{\text{eff}} l_r^2$. We note that the obtained values of $E_r^{\max}$ are significantly higher than the thermal energy (see Table S2), such that extracting the value of l_r from simulations performed at room temperature is a well justified approximation. All values for the abovementioned kinetic model bond rupture parameters appear in Table S2.

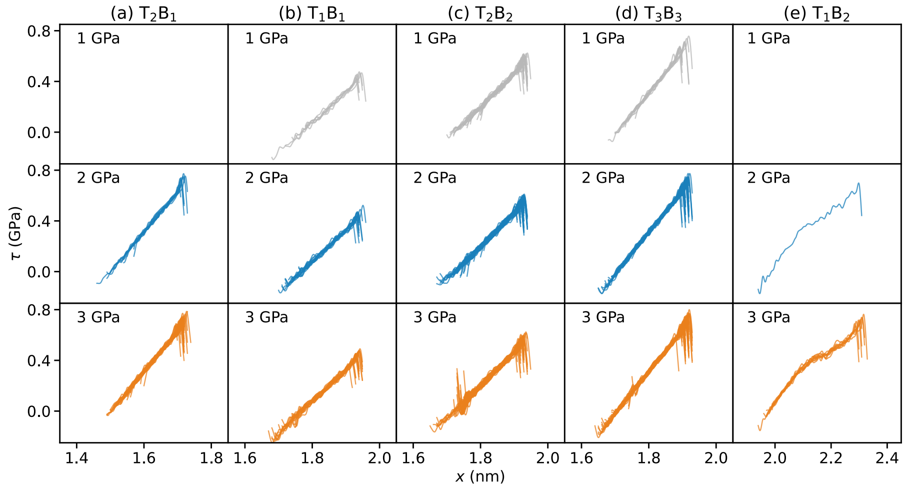

Fig. S7. Frictional shear stress traces for five bonded pairs: (a) T_2B_1; (b) T_1B_1; (c) T_2B_2; (d) T_3B_3; and (e) T_1B_2; obtained from reactive sliding dynamics simulation at a temperature of $T = 300$ K, a sliding velocity of $v = 10$ m/s, and under normal loads of $\sigma = 1$ GPa (top panels), 2 GPa (middle panels), and 3 GPa (bottom panels). The T_2B_1 and T_1B_2 traces under 1 GPa are not depicted as no interlayer bond formation was found to occur for these atomic pairs under the considered conditions.

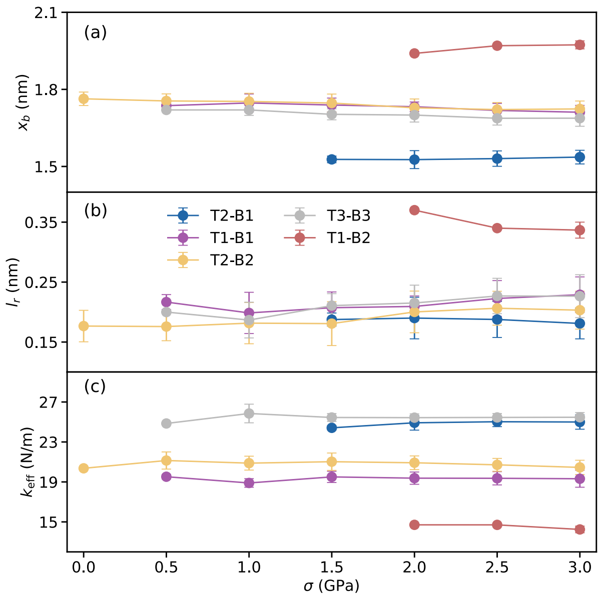

Fig. S8. Demonstration of the independence of (a) bond formation position (x_b), (b) rupture length (l_r), and (c) effective stiffness (k_{eff}) on the normal load (σ), as obtained from reactive sliding dynamics simulations at a temperature of $T = 300$ K and a sliding velocity of $v = 10$ m/s.

Table S2. Kinetic model interlayer bond rupture parameters (bond formation position (x_b), bond rupture length (l_r), load-free TEB (E_r^{max}), and effective bond stiffness (k_{eff})) for five bonded pairs.

Bonded pairs	T_2B_1	T_1B1	T_2B_2	T_3B_3	T_1B_2
x_b(nm)	1.53	1.73	1.74	1.70	1.96
l_r (Å)	1.9	2.1	1.9	2.1	3.5
E_r^{max} (eV)	2.70	2.76	2.32	3.53	5.53
k_{eff} (N/m)	24.8	19.3	20.8	25.4	14.6

References

Bitzek, E., Koskinen, P., Gähler, F., Moseler, M., Gumbsch, P., 2006. Structural relaxation made simple. Physical Review Letters 97, 170201.

Gao, X., Ouyang, W., Urbakh, M., Hod, O., 2021. Superlubric polycrystalline graphene interfaces. Nat. Commun. 12, 5694.

Jónsson, H., Mills, G., Jacobsen, K.W., 1998. Nudged elastic band method for finding minimum energy paths of transitions, Classical and quantum dynamics in condensed phase simulations. World Scientific, pp. 385-404.

Larsen, A.H., Mortensen, J.J., Blomqvist, J., Castelli, I.E., Christensen, R., Dułak, M., Friis, J., Groves, M.N., Hammer, B., Hargus, C., 2017. The atomic simulation environment—a Python library for working with atoms. Journal of Physics: Condensed Matter 29, 273002.

Muniz, A.R., Maroudas, D., 2012. Opening and tuning of band gap by the formation of diamond superlattices in twisted bilayer graphene. Physical Review B 86, 075404.

Schneider, T., Stoll, E., 1978. Molecular-dynamics study of a three-dimensional one-component model for distortive phase transitions. Physical Review B 17, 1302.

Ying, P., Natan, A., Hod, O., Urbakh, M., 2024. Effect of Interlayer Bonding on Superlubric Sliding of Graphene Contacts: A Machine-Learning Potential Study. ACS Nano 18, 10133-10141.
