# Supplementary material for: Chemifriction and Superlubricity: Friends or Foes?
Source: J Phys Chem Lett. 2025 Mar 13;16(11):2934–41. doi: 10.1021/acs.jpclett.5c00193 (PMC11931540; doi:10.1021/acs.jpclett.5c00193)
Supplement: Supplementary file 6 — jz5c00193_si_006.pdf [file jz5c00193_si_006.pdf]

jz-2025-00193k.R1

Name: Peer Review Information for "Chemifriction and Superlubricity: Friends or Foes?"

First Round of Reviewer Comments

Reviewer: 1

Comments to the Author

Interesting piece of work. Appreciable for its introduction of chemifriction, for the explicit simulation of friction in a graphene interface with a vacancy on each side, for the development of a probabilistic model rationalising the temperature, pressure, and velocity behaviour, and for the possible superlubricity-restoring healing process which is discovered under favourable conditions. The paper is well written, publishable in its present form.

Reviewer: 2

Comments to the Author

Ying and coworkers describe a bond-dynamics model for sliding of graphene. The idea is that under sufficient pressure, graphene may locally cold-weld (forming a diamond like structure) that is difficult to break and thereby increases the shear stress required for moving two graphene flakes with respect to each other, essentially killing superlubricity. The authors test the model with molecular dynamics calculations. Those calculations must capture covalent bond formation as well as the nonbonded (dispersion) force between the two graphene sheets. The authors accomplish this through a machine-learned potential that is specifically trained on DFT-calculations with Tkatchenko-Scheffler dispersion correction for the situation they study. The paper is well written and nicely illustrated. I recommend publication.

My only comment is that I am surprised the author did not dive into a discussion of the parameter alpha in their equation, which is the activation volume. There is lots of recent literature discussing the role of the activation volume, in particular in situations where normal stress changes the potential energy landscape. The authors give the value in units of eV/GPa (essentially camouflaging that it is a volume). The volume that they get is on the order of Angstroms^3. There may be a good reason not to discuss this (as there may be no clear interpretation of that parameters), but the authors may want to consider adding a statement on this.

Reviewer: 3

Comments to the Author

This is an excellent and novel contribution, showing that sliding on graphene can induce defects that actually reduce friction, in contrast to the nearly universal observation that defects increase friction. The authors explain the results clearly and convincingly using molecular dynamics simulations and an associated reduced order model to access low speed sliding, relevant for many experiments. I fully support publication, provided the following points can be addressed:

1. In the Abstract, the term "machine-learning molecular dynamics simulations" is potentially confusing; machine-learning is not the method used to obtain the results, but rather it is used to obtain the MD potentials. The wording should be changed to, eg "molecular dynamics simulations using potentials obtained with machine learning".

2. The abstract would be stronger if the nature of the pre-existing defect, and the shear-induced modified defect, were stated. Otherwise the Abstract has a somewhat 'black box' aspect regarding what is being considered. The word "defect" after all means anything from a vacancy to a dislocation to a surface. In this case, sliding causes an interlayer defect to "break" and transform into an in-plane defect that doesn't impede friction much. The idea is elegant and compelling; why not make it explicit in the abstract? Also in the first sentence "interlayer" would be clearer than "interfacial".

3. In the Introduction the authors should specify why they chose 9.43° twist angle. If for practical reasons, that's fine. The reader is left to wonder if there is something special about this angle physically.

4. The authors cite ref's 35-38 to support the idea that single atom vacancies can be unpassivated. Those papers are all TEM or STM studies conducted in high or ultrahigh vacuum. In air, there will be passivation. The authors should make clear the mechanism hinges on the lack of passivating species and that the references cited are for vacuum conditions. Otherwise readers may misinterpret the findings as being applicable to ambient conditions. This does not diminish the impact of the paper but it does help establish how the results could be validated with experiments.

5. Related to #4, is there any experimental evidence to support that such vacancies are common? Ref. 35 sees several in their particular graphene, but this does not mean it's in general expected to be a common occurrence. These vacancies have a high formation energy (7 eV according to some

calculations). The 5-7 Stone-Wales defect is likely more common. If this type of vacancy is not common, that's fine - but a comment regarding how common (or not) it is helps the reader understand the context better.

Author's Response to Peer Review Comments:

Reviewer #1

"Interesting piece of work. Appreciable for its introduction of chemifriction, for the explicit simulation of friction in a graphene interface with a vacancy on each side, for the development of a probabilistic model rationalising the temperature, pressure, and velocity behaviour, and for the possible superlubricity-restoring healing process which is discovered under favourable conditions. The paper is well written, publishable in its present form."

Response:

We thank the reviewer for her/his positive evaluation of our manuscript and for recommending it for publication in its present form.

Reviewer #2

"Ying and coworkers describe a bond-dynamics model for sliding of graphene. The idea is that under sufficient pressure, graphene may locally cold-weld (forming a diamond like structure) that is difficult to break and thereby increases the shear stress required for moving two graphene flakes with respect to each other, essentially killing superlubricity. The authors test the model with molecular dynamics calculations. Those calculations must capture covalent bond formation as well as the nonbonded (dispersion) force between the two graphene sheets. The authors accomplish this through a machinelearned potential that is specifically trained on DFT-calculations with Tkatchenko-Scheffler dispersion correction for the situation they study. The paper is well written and nicely illustrated. I recommend publication."

Response: We thank the reviewer for her/his positive evaluation of our manuscript and for recommending it for publication.

"My only comment is that I am surprised the author did not dive into a discussion of the parameter alpha in their equation, which is the activation volume. There is lots of recent literature discussing the role of the activation volume, in particular in situations where normal stress changes the potential energy landscape. The authors give the value in units of eV/GPa (essentially camouflaging that it is a volume). The volume that they get is on the order of Angstroms^3. There may be a good reason not to discuss this (as there may be no clear interpretation of that parameters), but the authors may want to consider adding a statement on this."

Response: We thank the reviewer for raising this important point. As the reviewer states, it is challenging to interpret the physical meaning of the activation volume parameter (α), as it may be associated with factors such as the nature of inter-vacancy bonding, the effective interaction range of each defect, and the defect density. Here, we follow a previous study (*Tribol. Lett.* **69**, 150 (2021)) by describing the activation volume as a measure of the response of chemical reactivity to external load, e.g., by linearly reducing the reaction barrier. Based on this understanding, our proposed probabilistic (phenomenological) model can accurately describe the bond formation probabilities estimated by reactive molecular dynamics simulations (see Fig. 2 of the main text). We also note that the activation volume that we derived, $0.048\ \mathrm{eV/GPa} \approx 1.974$ $\mathrm{\AA}^3$, corresponds well to values estimated for similar reaction processes studied in AFM experiments, such as the wear of amorphous hydrogenated carbon (*ACS Nano* **8**, 7027 (2014)) and crystalline Si tips (*Nat. Nanotechnol.* **8**, 108 (2013)) on diamond (see Table 1 of *Tribol. Lett.* **69**, 150 (2021)).

To convey this message better, we have modified the following sentence on page 6 of the revised manuscript:

"Furthermore, the TEB reduction is assumed to be linear with normal pressure $E_m(\sigma) = \alpha\sigma$ (46-48), where the activation volume, α, serves here as a measure of the response of chemical reactivity to external load.(43)." and on page 7, following the report of the fitted value of α:

"The value of α corresponds well to values estimated for similar reaction processes studied in AFM experiments, such as the wear of amorphous hydrogenated carbon (51) and crystalline Si tips (52) on diamond (see Table 1 of Ref. (43))."

Reviewer #3

"This is an excellent and novel contribution, showing that sliding on graphene can induce defects that actually reduce friction, in contrast to the nearly universal observation that defects increase friction. The authors explain the results clearly and convincingly using molecular dynamics simulations and an associated reduced order model to access low speed sliding, relevant for many experiments. I fully support publication, provided the following points can be addressed."

Response: We thank the reviewer for her/his positive evaluation of our manuscript and for recommending it for publication.

"1. In the Abstract, the term 'machine-learning molecular dynamics simulations' is potentially confusing; machine-learning is not the method used to obtain the results, but rather it is used to obtain the MD potentials. The wording should be changed to, eg 'molecular dynamics simulations using potentials obtained with machine learning'."

Response: We thank the reviewer for raising this point. To clarify, we have replaced the term "machine-learning molecular dynamics simulations" with "molecular dynamics simulations based on machine-learning potentials."

"2. The abstract would be stronger if the nature of the pre-existing defect, and the shear-induced modified defect, were stated. Otherwise the Abstract has a somewhat 'black box' aspect regarding what is being considered. The word 'defect' after all means anything from a vacancy to a dislocation to a surface. In this case, sliding causes an interlayer defect to 'break' and transform into an in-plane defect that doesn't impede friction much. The idea is elegant and compelling; why not make it explicit in the abstract? Also in the first sentence 'interlayer' would be clearer than 'interfacial'."

Response: We thank the reviewer for this useful suggestion. We have revised the first part of the abstract accordingly, which now reads as follows:

"The mechanisms underlying chemifriction, i.e., the contribution of interlayer bonding to friction in defected twisted graphene interfaces are revealed using fully atomistic molecular dynamics simulations based on machine-learning potentials. This involves stochastic events of consecutive bond formation and rupture between single vacancy defects that may enhance friction. A unique shearinduced interlayer atomic transfer healing mechanism is discovered that can be harnessed to design a run-in procedure to restore superlubric sliding."

"3. In the Introduction the authors should specify why they chose 9.43° twist angle. If for practical reasons, that's fine. The reader is left to wonder if there is something special about this angle physically. "

Response: We thank the reviewer for raising this point. Since we are interested in studying the effect of interlayer bonding on the superlubric nature of graphitic interfaces, we consider twist angle larger than ~7°, where the sliding friction becomes extremely low (*Phys. Rev. Lett.* **92**, 126101 (2004)). The specific choice of a 9.43° allows us to construct such a superlubric interface. One could, in principle, use larger twist angles. This, however, would require duplicating the (smaller) supercell and implanting defects in a small fraction of the supercells considered. The physi- and chemi-friction mechanisms in such case are still expected to hold. Hence, to avoid such complications, we opted to consider a twist angle that is large enough to support superlubric sliding yet small enough to include one defect in the single modeled supercell.

To address this point, we have added the following sentence in the model system description in the revised main text:

"The chosen twist angle is sufficiently large to provide a laterally periodic superlubric interface (in the absence of defects (39)) yet yielding large enough supercell dimensions to avoid spurious interactions between periodic images of the defects."

"4. The authors cite ref's 35-38 to support the idea that single atom vacancies can be unpassivated. Those papers are all TEM or STM studies conducted in high or ultrahigh vacuum. In air, there will be passivation. The authors should make clear the mechanism hinges on the lack of passivating species and that the references cited are for vacuum conditions. Otherwise readers may misinterpret the findings as being applicable to ambient conditions. This does not diminish the impact of the paper but it does help establish how the results could be validated with experiments.

5. Related to #4, is there any experimental evidence to support that such vacancies are common? Ref. 35 sees several in their particular graphene, but this does not mean it's in general expected to be a common occurrence. These vacancies have a high formation energy (7 eV according to some calculations). The 5-7 Stone-Wales defect is likely more common. If this type of vacancy is not common, that's fine - but a comment regarding how common (or not) it is helps the reader understand the context better. "

Response: We thank the reviewer for raising these important points. Following the reviewer's suggestions, we have provided in the revised text the following justification for our choice of model system:

“This fundamental model system allows us to isolate, identify, and study the basic mechanisms underlying chemi-friction and their interplay with physi-friction, while avoiding additional complexities arising from chemical passivation or other types of defects.” and added the following text to the revised summary:

“The frictional mechanism discovered herein based on unpassivated single-vacancy defect models may serve as a basis for understanding chemi-frictional behavior under more complex scenarios involving different types of defects, chemical passivation, and intercalants.”

Editorial formatting modifications

1. Please indicate the corresponding author(s) with an asterisk in the author list on the manuscript title page.

Response: Done.

2. An email address is required for each corresponding author identified on the manuscript file. Please add the corresponding author email(s) to any page of the manuscript.

Response: Done.

3. Include a TOC graphic illustrating the significance of the paper. For TOC guidelines and size requirements, please see the following link: https://pubsapp.acs.org/paragonplus/submission/toc_abstract_graphics_guidelines.pdf?
Please label the TOC graphic as “TOC Graphic”. A caption describing the TOC graphic is not needed and will not be used.

Response: Done.

4. Please remove the section headings from your manuscript file, e.g., Introduction, Results and Discussion, Conclusion. Experimental section headings as well as paragraph headings are okay.

Response: Done.

5. If the manuscript is accompanied by any Supporting Information for Publication, the manuscript should contain a brief, nonsentence description of the actual contents of each Supporting Information file. This description should be labeled Supporting Information and should appear directly before the Acknowledgment and Reference sections. The appropriate

format is as follows: Supporting Information. Brief statement in nonsentence format of the contents of the material supplied as Supporting Information.

Response: Done.

6. Please include author names, article titles, journal name, publication year, and at least the first page number for each reference citation for the following incomplete journal references: 32, 33, 52.

Response: Done.
